# Where Is the Parent’s Voice? A Meta-Synthesis of Parental Experiences of Video Feedback Parenting Interventions

**DOI:** 10.1007/s10567-025-00514-w

**Published:** 2025-02-01

**Authors:** Ming Wai Wan, Tarendeep. K. Johal, Anja Wittkowski

**Affiliations:** 1 Division of Psychology and Mental Health, School of Health Sciences, Faculty of Biology, Medicine and Health, The University of Manchester, Manchester Academic Health Science Centre, Jean McFarlane Building, Oxford Road, Manchester, M13 9PL UK; 2 Greater Manchester Mental Health NHS Foundation Trust, Manchester, UK

**Keywords:** Parent–child, Video-aided guidance, Video feedback, Parenting program, Lived experience, Attachment-based, Interviews, Mother-infant, Qualitative

## Abstract

Video-aided feedback (VF) is a well-evidenced intervention technique to enhance the relationship between a parent and their young child. While parental acceptability is foundational to engagement and intervention efficacy, the parent’s perspective is only now emerging as a valued consideration when evaluating VF-based interventions. This systematic review metasynthesised qualitative research on the experiences of primary caregivers with a young child (primarily 0–30 months) of participating in a VF parenting intervention. A search of nine databases yielded 17 studies (10 published since 2020) involving parents who had participated in range of VF-focussed programmes. Thematic synthesis identified six themes: (1) Getting past the fear and discomfort: Being ‘good enough’ and ‘doing the right thing’; (2) The power of video: “I had never really noticed that before‟ (with two subthemes: video as validation and for seeing child behavioural intentionality; video as an agent for change); (3) The practitioner’s skill and role in creating a safe space; (4) The approach was too intangible, inflexible, positive, unclear; (5) When the intervention is over: Positive change and generalisation; (6) Parental engagement and involvement: Barriers and enhancements. While most parents reported experiencing a range of interpersonal and intrapersonal benefits from taking part in a VF parenting intervention, having to overcome initial strong negative and uncomfortable feelings were an important part of the journey. Some parents could not see the value of the approach or did not perceive the intervention to meet their needs. Insights into parental experience are complementary to outcome-based evaluations. However, biased design (e.g., only one study included intervention non-completers) and variable study quality need addressing in future studies. Implications for practice are discussed.

## Introduction

A child’s relationship with their primary caregiver is considered the most important relationship an individual will experience, and dimensions of this relationship in the early years predict a range of child developmental outcomes (e.g., Deans, 2020; Rocha et al., 2020). However, various specific vulnerabilities can challenge this relationship, including parent (e.g., mental health; Center on the Developing Child at Harvard University, 2009; Wan & Green, 2009) and child conditions (e.g., behavioural difficulties; Schneider et al., 2022, and neurodevelopmental conditions; Wan et al., 2019). Frame-by-frame film analysis was first used by Stern (1971) to study mother-infant interactions, and psychological interventions for parents were later developed incorporating video-aided feedback (VF) guidance as the core or a key component to enhance the parent–child relationship or attachment for a range of participant groups and conditions.

VF-based parenting interventions tend to focus on the relational qualities between parents of infants and young children (e.g., Bakermans-Kranenburg et al., 2003; Newton et al., 2022; O’Hara et al., 2019), in recognition of the critical importance of the first 2–3 years for the child’s subsequent outcomes (Jeong et al., 2021). While intervention characteristics and longer-term goals may vary, the basic VF process is similar: After the parent is filmed interacting with their child, they are invited to co-view and reflect on the video content (entirely or post-edit) in detailed discussion with a trained practitioner or clinician (Balldin et al., 2018). The power of video lies in the parent’s observation of themselves and their child ‘from the outside’ of themselves, combined with the triangulating space created by the practitioner in which reflective discussions are facilitated. By being invited to comment on what they see in the video, the parent is positioned as the expert of their child (Juffer et al., 2023). Their video observations facilitate or enhance the parent’s realisation of their child as a separate ‘mentalising’ being, also known as parental reflective functioning, which is closely tied to parental sensitivity (Beebe, 2003; Jones, 2006). By repeating the VF process over several sessions, each time using newly collated parent–child interaction videos, the parent’s further noticing and realisations pave the way to quick emotional, cognitive and behavioural shifts in how they relate to and interact with their child.

Bakermans-Kranenburg et al.’s (2003) seminal meta-analysis of attachment-focussed interventions found that behavioural interventions with VF were more effective than those without VF in improving attachment and relational outcomes. The benefits of VF-based intervention for enhancing observed caregiver-infant interaction – usually the key proximal therapeutic target—and a range of other parenting behavioural and child outcomes are highlighted by other reviews (Balldin et al., 2018; Fukkink, 2008; O’Hara et al., 2019). Notably, Balldin et al.’s (2018) review found that interventions with a VF component enhanced positive parent–child interaction (especially maternal sensitivity) relative to treatment as usual, with generally small to moderate effects, although several studies showed large effect sizes. O’Hara et al.’s (2019) meta-analysis focussed solely on VF interventions and found a moderate positive effect across twenty-two studies on maternal sensitivity compared with controls from post-intervention to six-month follow-up, which they noted were similar to the effects of other interventions and favourable to home visitation programmes. However, their compelling translation of a moderate effect size (“if 10,000 parents were to receive a video‐feedback intervention, around 1100 of them would benefit”) suggests that parents vary widely in how they experience VF interventions.

While the evidence is generally positive, few (high quality) studies of VF interventions have involved parents experiencing perinatal mental health difficulties or father participants, and there is little evidence to suggest that VF interventions are helpful for reducing parental stress and anxiety (O’Hara et al., 2019). Fukkink’s (2008) meta-analysis of VF interventions suggests that parental perceptions (e.g., self-confidence, stress) are much more resistant to change in high-risk parent groups, including samples with depression, poverty, single parenthood and adult insecure attachment.

It is particularly important to understand how parents *experience* the VF process because VF is usually co-constructed between the practitioner and parent (and much more rarely, practitioner-directed). As such, success of this client-centred process requires parental engagement, quite intense levels of proactive involvement within and often between sessions, and reflective discussions in collaboration with the practitioner. What parents think and feel about participating in this process is critical to the formation of a positive therapeutic alliance and commitment to change. The few studies that incorporated parent survey feedback as part of feasibility and acceptability testing involved very small samples and provided limited scope. For example, fathers (Lawrence et al., 2013) and parents with a toddler with autism (Klein et al., 2021) rated highly that they benefitted from learning about their child, and the latter group reported no problem operating the camera or recording the videos.

Practitioners’ ideas about how parents experiences VF interventions may differ greatly from parents’ actual experiences. Doria et al.’s (2014) quantitative content analysis of mothers’ and practitioners’ interviews of participating in Video Interactive Guidance found that the mothers’ most commonly reported ‘active ingredients’ of VF were self-reflection (20%) and the video itself (17.5%), neither of which were reported by practitioners. By contrast, the practitioners more often mentioned supported co-exploration (20.5%) and the positive/success focus (15.4%) as the critical components of the success of VF, neither of which were discussed in the parent interviews. VF can be an unfamiliar process for parents due to the use of video and an often non-directive approach, although more directive coaching is sometimes used. Parents may feel low and lacking in parental confidence (Vik & Rohde, 2014) and anxious and self-conscious about being video recorded and watching themselves (Domoney et al., 2015).

To date, no review of VF parenting interventions has considered parents’ lived experiences of participation. While Butler et al. (2020) reviewed 26 studies of parents’ qualitative experiences with parenting interventions, none involved VF programmes in the early years. This review thematically metasynthesised qualitative studies of parents’ experiences in VF interventions, which focussed on enhancing the parent–child relationship in the earliest years, a main target of VF parenting interventions (Bakermans-Kranenburg et al., 2003). Parents with a child aged 0–30 months were our target sample group, though studies with children up to five years were included if the mean age was 0–30 months. Our objective was to understand parents’ experiences in VF interventions and to capture positive and negative experiences across various participant groups, contexts (community and clinical), and programs, highlighting what they valued, found challenging, and recommended.

## Method

### Design

A qualitative thematic metasynthesis was conducted. Qualitative metasynthesis is a methodological technique to accumulate knowledge from qualitative studies by systematically reviewing findings reported across multiple studies in a particular field to generate new interpretations and insights (Paterson et al., 2009), which was adapted from thematic analysis. Since there has been increasing interest in considering the lived experiences of service users, this approach was considered appropriate as we were particularly interested in interpreting, evaluating and presenting commonalities in experience in different studies utilising a similar overall intervention approach. Furthermore, the approach is useful when the findings of primary qualitative studies are under-utilized (Wa-Mbaleka, 2020), so a synthesis can highlight advancements needed in the field. We selected a thematic approach to focus on understanding common themes in experience across studies and interventions to understand common motivators and challenges. After identifying the research question, the metasynthesis involved four phases: (1) developing and refining an appropriate and feasible search strategy informed by running preliminary scoping searches in an iterative process; (2) searching for relevant studies in a systematic way adhering to predefined parameters and eligibility criteria; (3) extracting text content from eligible studies, specifically, the method section (to tabulate) and results section (descriptions of the authors’ findings and selected quotes, to synthesise), (4) coding the results text content to find emergent themes across findings, and generating new insights from the studies. After completing the first phase, the protocol for this meta-synthesis was registered with PROSPERO (Ref: CRD42022374587) on 13/12/2022.

### Search Strategy

**Fig. 1:**
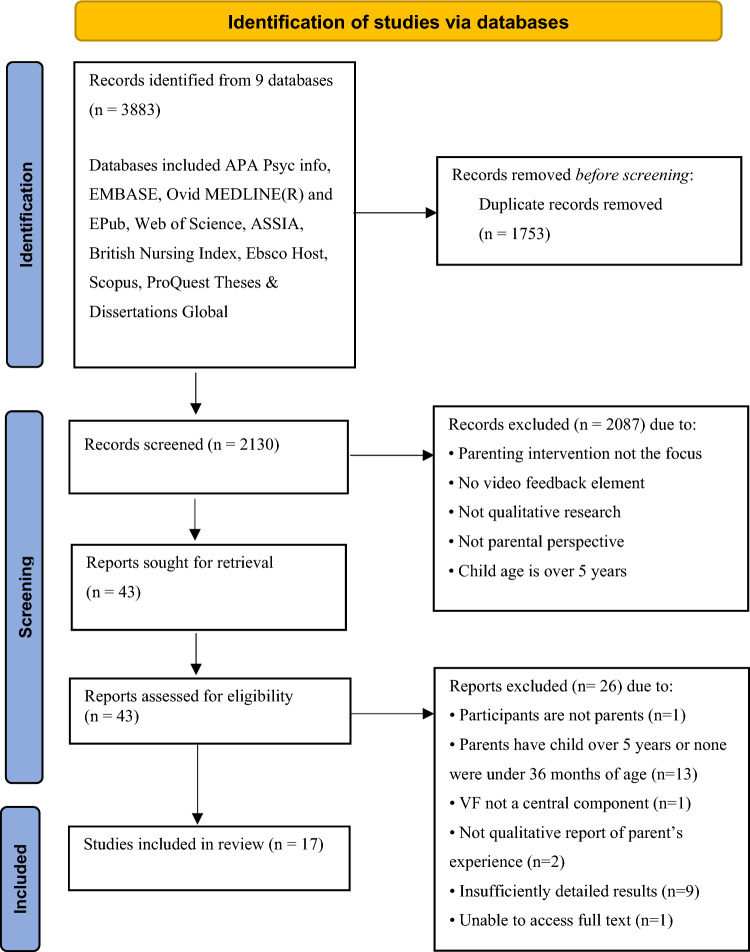
PRISMA flow diagram

The search strategy was developed in consultation with the [source withheld] library service and informed by PICOS (Methley et al., 2014). Nine databases (Fig. 1) were searched in December 2022 for articles published from inception based on the following search terms (in title, abstract or keywords): Population: parent* OR infan* OR “mother-infant” OR maternal OR baby; Intervention: Video OR VIG OR VIPP-SD; Comparison, Outcome and Sample: Qualitative OR view* OR experience* OR pilot OR feasibility OR acceptability or interview* OR “mixed methods” Modality: Intervention OR program* OR treatment OR therap*. The review’s focus on interventions that enhance the parent–child relationship is reflected in the population search terms, which is a main age group of focus in VF parenting interventions (Bakermans-Kranenburg et al., 2003).

The second author (TJ) screened the abstracts of 2130 unique papers (Fig. 1) based on the inclusion criteria below, identifying 43 potentially eligible papers for full assessment, managed in EndNote (Clarivate Analytics UK Ltd Version 20, 2020). TJ and an independent assessor assessed the full text of studies for eligibility (n = 43), showing substantial agreement (86%; Kappa 0.72); the inclusion criteria and research aims guided decisions to reach consensus over disagreements.

### Inclusion and Exclusion Criteria

English language studies (peer reviewed papers and theses) were included that report qualitatively the experiences and views of primary caregivers (referred to as ‘parents’ for convenience) with a child of 0–30 months of age about their experiences of taking part in a parenting intervention that incorporated VF (of the caregiver’s own interactions with the child) as the key component. However, as qualitative studies of parents’ experiences of VF interventions were expected to be relatively novel, an inclusive approach was taken, which allowed studies including broader age ranges up to five years of age to still be included in the review as long as the mean age was within the target age range of 0–30 months. All study types (e.g., case reports, randomised control trials) were eligible if relevant qualitative (i.e. text-based) data could be extracted. Papers were excluded if: the intervention had no substantial VF component involving caregiver-infant interaction, samples included any caregivers with a target child aged > 5 years, or the study did not involve obtaining caregivers’ lived experiences of the VF intervention (e.g., descriptions on understanding of the intervention).

### Data Extraction and Analysis

From each study, all sections of the paper were carefully checked from which key study characteristics were extracted (authors and country, sample (number, type of caregiver), key sample characteristics as basis for targeting intervention, child age, intervention programme, intervention context (number of sessions, setting), and qualitative data collection and analytic method). Studies were tabulated by intervention programme, where possible. Corresponding authors were contacted if key sample information was missing. The extraction table was completed by TJ and cross-checked with papers independently by MWW to ensure accuracy, and study and intervention characteristics were summarised narratively.

An interpretive thematic analysis was conducted (Thomas & Harden, 2008) to identify common themes across multiple qualitative studies. Firstly, the qualitative results sections of papers for review were compiled, including quotations from parents. Author interpretations from the studies were also extracted to inform the analysis, although quotations were prioritised for theme development. Irrelevant sections were removed (e.g., content around specific non-VF aspects, quantitative data). The second (TJ) and first (MWW) author completed the thematic synthesis in three stages: 1) Extracted data were read several times and familiarisation before line-by-line coding led by TJ; (2) Codes were organised into related areas through an iterative and inductive process to create descriptive themes that emerged organically from the data, led by TJ and closely supported by MWW; and 3) Using the descriptive themes, analytical themes and (where appropriate) subthemes were created to address the review objectives. Researchers made inferences when interpreting consistent and inconsistent themes across papers, and the final themes were scrutinised as a team to ensure they were plausible, coherent and appropriately derived from the data. Quotes were selected to reflect the breadth of sample characteristics, as appropriate, and a schematic diagram was developed based on the derived themes to best represent parental experiences of participating in a VF intervention as per the primary review objective.

### Methodological Quality Assessment

While a standard quality appraisal tool (Critical Appraisal Skills Programme, 2018) was initially employed, there were issues with over-estimating study quality and assessing pilot feasibility studies. After careful scrutiny of the studies, we applied ten objective criteria for considering completeness of reporting, likely saturation and rigor, based on the reporting or presence of: (1) 7 + participants; (2) basic intervention context information (where, number of sessions); (3) Intervention non-completers recruited; (4) basic description of what intervention participants (activities, practitioner approach); (5) data collection independent of the practitioner; (6) what participants were asked; (7) formal analytic approach; (8) analytic details and/or reflections; (9) credibility checks or collaborative coding; (10) publication in a peer reviewed journal. A score of 1 was assigned to each study based on assigning a point to each criterion fulfilled (half point for partial fulfilment).

## Results

### Overview of Studies

Of the 2130 unique papers identified, seventeen studies were synthesised. Studies were published between 2005 and 2022. However, with ten of the seventeen identified studies published since 2021 (4 studies in 2021, 6 studies in 2022), parents’ experiences of VF interventions is a topic largely of recent academic interest. Four studies were from doctoral theses. Data collection of parent perspectives were gained through semi-structured interview (14 studies), focus groups (2 studies), or open interview question (1 study). In terms of analytic methodology, thematic analysis was most favoured, but phenomenological and simple descriptive approaches were also taken (Table 1). Length of interviews and depth of analysis varied widely. Two studies involved the same team, sample, interview topics and transcript length (average/range) with the apparent difference being the mentalisation focus of one study (Simhan et al., 2021a, 2021b). Both studies were retained because their findings were considered sufficiently different (albeit with overlap).

**Table 1:** Study and intervention characteristics

	Author/s, country	Sample	Sample characteristics	Child age	Programme	Sessions and setting	Methods
*Marte Meo*							
1	Gill et al. (2019), Norway	2M, 2F	P: MH difficulties	0-20m	Marte Meo	2–8 in clinic	I, phen
2	Simhan et al. (2021a), Norway	11M, 1F	P: Chronic MH difficulties	2-27m	Marte Meo	3–7 at home	I, TA
3	Simhan et al. (2021b), Norway	11M, 1F	P: Chronic MH difficulties	2-30m	Marte Meo	3 + , setting not stated	I, TA
4	Vik and Hafting (2009), Norway	15M	M: Mid + depression scores	6-24m	Marte Meo	Not stated (recruited from health centre)	I (pre/post), phen
*Video Interaction Guidance (VIG)*							
5	Chakkalackal et al. (2021), UK	6P	P: Socially disadvantaged	< 12m	VIG	6 at home	Telephone I, TA
6	Elliott (2020), UK	3M	Adolescent mothers	5-18m	VIG	3 at home or CC	I, TA
7	Robinson (2020), UK	3M	C: Deaf / hard of hearing	22-28m	VIG	3 at home	I, TA
*Video Intervention to Promote Positive Parenting (VIPP)*							
8	Cleary et al. (2022), Australia	13M	Obstetric / antenatal services	Up to 8m	iBASIS-VIPP	7–10 at home	I, descriptions
9	Dugmore et al. (2022), UK	5P	Adoptive families	1–5 years	VIPP-FP	7 at home	Telephone I, TA
10	Iles et al. (2017), UK	6M, 5F	C: Behaviour difficulties	10- 24m	VIPP-Co	6 at home	I / qur, TA
11	Williams (2017), UK	14F, 11M	C: Behaviour difficulties	12-36m	VIPP	6 at home	I, TA
*Other VF interventions*							
12	Amsbary et al. (2021), US	6M	C: Features of possible autism	Under 30m	PII with VF	Not stated, at home	I, TA
13	Auge (2005), UK	6M	M: Low maternal sensitivity	7-14m	Brief VF	1–4 at CC	I, GT
14	Bergsund et al. (2024), Norway	9M, 1F	Child welfare services	6-24m	ABC	10 at home	SWOT-based I, FA
15	O’Leary et al. (2022), US	9M, 2G	NAm communities	1–4 years	PFR	10 at home	FG, themes
16	Ousley et al. (2022), US	3M	C: Communication delay	2–5 years	VF coaching	4–6 via video conferencing	Qur; description with quotes
17	Senent-Capuz et al. (2022), Spain	7M, 5F	C: Language impairments	18-40m	ITTTT	8 group sessions in clinic; 3 at home	FG, content analysis

*ABC* Attachment and bio-behavioral catch-up, *CC* community centre, *F* fathers, *FA* framework analysis, *FG* focus groups, *G* grandmothers, *GT* grounded theory, *I* interview, *ITTTT* it takes two to talk (Hanen), *IPA* interpretive phenomenological approach, M mothers, *MH* mental health, *NAM* Native American, *P* parents, *PFR* promoting first relationships, *Phen* phenomenological, *m* months, *PII* parent-implemented intervention, *Qur* questionnaire, *TA* thematic analysis, *VF* video feedback

#### Sample Characteristics

Studies represented the views of 157 parents (sample size range: 3–25 parents) from five countries (Australia, Norway, Spain, UK, and US; Table 1). However, small samples were common; eight studies had six or fewer participants. Samples comprised of parents (8 of mothers or fathers, 7 of mothers, 2 of mothers and grandmothers). In terms of the parent’s target child for the intervention, 15 studies involved parents with a child in the 0–24 month range, while 9 studies covered some (2 + months) of the 23–35 month range. We included four studies that focussed on parents of slightly older children (36 month to 5 years) because they also included parents of children aged 2 years. Many studies included parents of children across more than one age group.

Studies tended to focus on intervention feasibility when implemented in or adapted for specific groups (e.g., adolescent mothers, children at risk of behavioural problems, elevated likelihood of autism, co-parents), contexts (e.g., socioeconomic disadvantage, self-recorded videos, implementation by child welfare services), or communities (e.g., Native American, specific countries). Six studies recruited based on parent characteristics, including parental mental health difficulties (4 studies), adolescent motherhood (1 study), and ‘severe emotional disturbances’ within mother-infant interaction (1 study). Seven studies recruited on child characteristics: language and communication concerns (2 studies), behavioural difficulties (2 studies), hearing difficulties (1 study), and elevated likelihood of autism (1 study). Four studies recruited based on family characteristics (one of each): adoption, child welfare involvement, Native American community, and social disadvantage. A general community sample came from obstetric and antenatal services. Most studies recruited from health settings (e.g., health centres, maternity wards, children’s centres), followed by community settings (childcare settings, charitable organisations).

#### Intervention Characteristics and Context

Three VF programmes dominated (Table 1). Four studies involved *Video Feedback Intervention to Promote Positive Parenting* (VIPP; Juffer et al., 2008), including adaptations for co-parenting, adoptive families, and infants at elevated likelihood of autism but employed in a general population sample. Three of these studies took place in the UK and the other in Australia. A further four studies, all Norwegian, involved *Marte Meo* (Aarts, 2008). Three studies, all UK-based, involved *Video Interaction Guidance* (VIG; Kennedy, 2011). The rest involved one study each of: *Attachment and Bio-Behavioral Catch-up* (ABC; Dozier & Bernard, 2019), *It Takes Two to Talk Hanen Program* (Pepper & Weitzman, 2004), a *Parent-Implemented Intervention* including VF (and with non-VF components; e.g., reviewing video modelling examples) (Schwertz et al., unpublished manual cited in Amsbary et al., 2021); *Promoting First Relationships* (Kelly et al., 2016), a brief VF programme in Surestart (Auge, 2005), and VF coaching via videoconferencing (Ousley et al., 2022).

Most interventions centred around VF; some were manualised and structured around specific themes as sessions progressed (e.g., VIPP), though some were not manualised (e.g., VIG) or the degree of structure was unclear. Sessions mostly involved an individual practitioner who filmed the parent–child interactions and facilitated VF. In some interventions, the practitioner was very much a facilitator (discussion is parent-led and exploratory) and in others, more of a coach (discussion is practitioner-led and instructive). For ease, we refer to this trained individual as a ‘practitioner’, irrespective of approach and context. A few studies involved additional components; e.g., reviewing videos of others in a peer group (Senent-Capuz et al., 2022) and reviewing video examples of modelling (Amsbary et al., 2021). Our analysis excluded coverage of non-VF areas.

Interventions ranged between “1–3 sessions” and 10 sessions, with 4–6 sessions being most common. For 11 studies, sessions took place largely or completely in the home, or that option was provided, while the remainder were situated in a clinic or community setting, or online (Table 1). Primary targets of the interventions included: enhancing mother–child interaction (e.g., sensitivity, attunement, attachment, capacity to treat the child as a psychological agent), decreasing maternal depressive symptoms, promoting parental bonding and positive relationships, and enhancing social communication skills (language, joint attention, and play skills).

#### Quality of Included Studies

Study quality varied considerably (Table 2). In Marte Meo and VIG studies, the practitioner also served as the interviewer or it was unclear if this was the case, and little information was provided on the interview questions. Moreover, VIG studies were small pilot or feasibility studies, reported mainly in PhD theses, with no reported credibility checks or collaborative analysis, and Marte Meo studies tended to describe little of the intervention. The most robust study, which also included intervention non-completers, came from O’Leary et al.’s (2022) study of Native Americans. On the other hand, Ousley et al. (2022) included a sample of three mothers and only one open-ended question. Bergsund et al. (2024) incorporated parental and practitioner views in one analysis, which were sometimes hard to disentangle. Higher quality studies were prioritised in the narrative synthesis.

**Table 2:** Quality indicators of methodology and reporting across studies (1 = present; 0 = absent; NR = not reported)

Author/s, year	Sample	1	2	3	4	5	6	7	8	9	10	Score
*Marte Meo*												
Gill et al. (2019)	P, PMH	0	0	1	½	0	½	1	1	0	1	5
Simhan et al. (2021a)*	P, PMH	1	0	1	½	NR	1	1	1	1	1	7.5
Simhan et al. (2021b)*	P, PMH	1	0	1	½	NR	1	1	1	1	1	7.5
Vik and Hafting (2009)	M, MMH	1	0	1	0	0	0	1	1	1	1	6
*VIG*												
Chakkalackal et al. (2021)	P, SD	0	0	1	½	0	½	1	½	0	1	4.5
Elliott (2020)	M, M-adol	0	0	1	1	0	0	1	1	0	0	4
Robinson (2020)	M, CH	0	0	1	1	0	1	1	1	0	0	5
*VIPP*												
Cleary et al. (2022)	M, Comm	1	0	1	1	1	½	½	0	0	1	6
Dugmore et al. (2022)	P, Adopt	0	0	1	1	1	½	½	½	0	1	5.5
Iles et al. (2017)	P, CB	1	0	1	1	1	½	1	1	1	1	8.5
Williams (2017)	P, CB	1	0	1	½	0	1	1	1	1	0	6.5
*Other*												
Amsbary et al. (2021)	M, C-EL	0	0	½	½	1	1	1	1	1	1	7
Auge (2005)	M, MS	0	0	1	1	1	1	1	1	0	0	6
Bergsund et al. (2024)	P, Welf	1	0	1	½	1	1	1	1	1	1	8.5
O’Leary et al. (2022)	M G, NAm	1	1	1	1	1	1	1	1	1	1	10
Ousley et al. (2022)	M, CC	0	0	1	1	1	0	0	0	0	0	3
Senent-Capuz et al. (2022)	P, CC	1	0	1	½	NR	0	1	1	1	1	6.5

Study characteristic: (1) Sample of 7 or more participants. (2) Study included intervention non-completers. (3) Intervention context reported (e.g., number of sessions, where it took place) (1/2 = partial). (4) Intervention activities and practitioner’s approach described (1/2 = partial). (5) Qualitative data collection independent of the intervention practitioner; (6) Interviewer questions or topics outlined (1/2 = partial). (7) Formal analytic approach reported (1/2 = partial / suggested). 8. Any analytic details and reflection reported (1/2 partial). 9. Credibility checks completed or collaborative coding reported. 10. Publication in a peer reviewed journal (rather than thesis)

*Same sample; Group: Sample grouping / nature of ‘risk’ (*Adopt* Adoptive families, *CB* child language and behaviour, *CC*: child communication, *C-EL* child with elevated likelihood of autism, *CH* child hearing, *Comm* community sample, *G* grandparents, *M* mothers, *M-adol* adolescent mothers, *MMH* maternal mental health, *MS* maternal sensitivity, *NAm* Native American families, *P* parents, *PMH* parental mental health, *SD* socioeconomic disadvantage, *Welf* child welfare involvement)

### Study Themes

Six main themes emerged from the thematic synthesis: (1) Getting past the fear and discomfort: Being ‘good enough’ and ‘doing the right thing’; (2) The power of video: “*I had never really noticed that before*‟ (with two subthemes: Video as a tool of validation and for seeing behavioural intentionality; Video as an agent for change); (3) The practitioner’s skill and role in creating a safe space; (4) The approach was too intangible, inflexible, positive, unclear; (5) When the intervention is over: Positive change and generalisation; (6) Parental engagement and involvement: Barriers and enhancements.

**Fig. 2:**
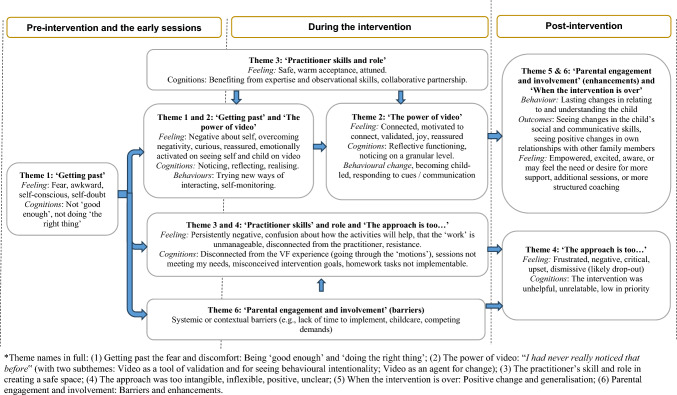
Schematic diagram of the parents’ chronological journey through the VF intervention process, based on the review themes. Theme names in full: (1) Getting past the fear and discomfort: Being ‘good enough’ and ‘doing the right thing’; (2) The power of video: “*I had never really noticed that before*‟ (with two subthemes: Video as a tool of validation and for seeing behavioural intentionality; Video as an agent for change); (3) The practitioner’s skill and role in creating a safe space; (4) The approach was too intangible, inflexible, positive, unclear; (5) When the intervention is over: Positive change and generalisation; (6) Parental engagement and involvement: Barriers and enhancements

The themes are schematically represented in Fig. 2, which illustrates the parent’s journey through the VF intervention process (pre-intervention and early sessions, during the intervention, post-intervention), reflecting the parents’ feelings and thoughts that were prominent across parent groups. The dominating narrative describes a journey that is positive, and for many transformative, with both interpersonal and intrapersonal benefits (Themes 2 and 5), once they became accustomed to the VF process (Theme 1), facilitated by the practitioner’s qualities (Theme 3). However, some parents struggled with the non-directive, client-led or strengths-based nature of the approach (Theme 4), some felt prevented from engaging in the process due to various barriers (Theme 6) and, rarely, a parent struggled to connect with the VF process or practitioner (Theme 2). While experiences were rarely wholly positive or negative, the challenges reported by parents could be understood as having substantial impacts on intervention engagement and involvement. We will discuss each theme in turn.

#### Theme 1: Getting Past the Fear and Discomfort: *Being ‘Good Enough’ and ‘Doing the Right Thing’*

Parents commonly experienced negative emotions prior to and during the intervention process, including self-doubt, guilt and anxiety, particularly in terms of being a ‘good enough’ parent, and ‘doing the right thing’. Across many studies, however, perceived inadequacy in parenting ability motivated participation. New parents described parenting as *“really scary”* (father, Williams, 2017) and expressed uncertainty about how to parent: *“Am I not looking after him right, ‘Am I playing with him right?’, cus no one really knows when it’s your first child as well, how you are supposed to play with them.”* (adolescent mother; Elliott, 2020). Intense feelings of inadequacy fed into one parent’s reluctance to participate in the intervention. However, some said they had participated out of fear of (perceived) consequences from services if they had refused: “*So, this is not about trust, this is about an enormous fear that they could use that against me later [if they did not participate]…*” (parents with chronic mental health difficulties; Simhan et al.,. 2021b).

Irrespective of the reasons for participating, parents feared being judged on their parenting skills and practices, especially in earlier intervention sessions. Both mothers and fathers reported feeling uncomfortable, self-conscious and awkward about the VF process. Perhaps most illustratively, several studies labelled this theme, ‘under the spotlight’. Parents felt pressure to impress and show the best ‘version’ of themselves: *“I wanted to look good in front of professionals… every parent wants to look like they’re the perfect parent… and just doing everything right”* (parent with low sensitivity; Auge, 2005). Some found the camera or practitioner who was filming distracting, and some judged their recordings as unrepresentative of their usual interactions. Some feared that the VF process meant viewing negative aspects of their interactions and evidencing they were *“doing it wrong”* (Iles et al., 2017), fuelling their sense of inadequacy. In O’Leary et al. (2022), both intervention completers and non-completers from Native American communities expressed discomfort in front of the camera and a desire to have more instructions during videotaped episodes.

Many parents reported that these uncomfortable feelings dissipated with time and by building a positive relationship with the practitioner. As the sessions progressed, they felt more comfortable, the camera became less distracting, and they could focus on the interaction: “*…the more you see the videos back the more you appreciate it’s not about you being filmed, it’s more about watching [infant’s name] and his reactions about things*’ (parent with difficulties managing their child’s behaviour; Iles et al., 2017); “*so after that initial sort of embarrassment it is, it is really just quite fascinating, looking at your baby from a different angle*” (mother with low sensitivity; Auge, 2005). Some parents remarked overcoming their discomfort after the first session: “*Honestly, after the first visit, it was easy for me because I didn’t feel judged, so that made it 100% easier to be open to more of like, ok they’re not judging me. That first visit did it for me. It kind of washed away my insecurity*” (Native American caregiver; O’Leary et al., 2022).

#### Theme 2: The Power of Video: *“I Had Never Really Noticed That Before”*

“Film” or “video” commonly appeared in theme and subtheme names across studies that identified themes, referring to the benefits or power of film. Across most studies, watching videos of parent–child interaction guided by a practitioner was discussed in a positive way. Many parents expressed a strong sense of enhanced awareness and validation or reassurance (subtheme 1), and some parents described this internal process being accompanied with experiencing behavioural change (subtheme 2).

##### Subtheme 1: Video as Validation and for Seeing Their Child’s Behavioural Intentionality

By enabling parents to see their child’s cues and own responses more clearly *“from the outside”* (Vik & Hafting, 2009), many described the reviewing of videos as empowering, validating and informative: “*watching it [video] back, you do sort of look at yourself in a different light and actually see how you really are…*” (mother with very low sensitivity; Auge, 2005). Parents valued the here-and-now and pausing elements of using video. This contrasted with how quickly things happen in real time along with the reality of daily distractions. Parents reported increased awareness of the child’s behaviours toward them in most studies. Especially among parents who had infants, many had unexpected realisations about their infant’s intentionality and attempts to communicate with them: “*That one sees things one has seen before, but it is more visible (…) that he communicates more than I thought (…) it has made me more attentive towards looking for minor things*” (parent with mental health difficulties; Gill et al., 2019).

Mothers with mental health difficulties particularly remarked on seeing their child’s gaze on video and the positive meaning it gave them, which they did not see or feel in real time: “*It has opened my eyes in a completely different manner, and I am now aware of things that I didn’t notice earlier, especially the way he looks at me”* (mother with moderately raised depression scores; Vik & Hafting, 2009). Similarly, in Simhan et al.’s (2021a) study of four parents with recurrent mental health difficulties, the authors state that: “*Most parents described that it was their infant’s gaze that first captured their attention and engendered the change process […] Their infants’ contact initiatives and attempts to continue these connections arrested the parents and made them aware of how their infants sought them, waited for their attention and expressed delight in them*”.

For parents who had struggled to bond, the video was proof of their own and their child’s attempts to develop a bond and mutual enjoyment: *“When you look back at the videos, I could actually see her looking at me for approval, I could see she was trying to develop a bond and it was enough to keep me going”* (adoptive parent; Dugmore et al., 2022). Faced with the ‘reality’ that was presented in the video, the parent had to resolve what they see with their own perceptions of themselves, which were often affected by their mental health: “*It was an especially large gap between the film and how I myself thought it looked*” (mother with depressive symptoms and psychosocial risk; Gill et al., 2019); “*When you are depressed you make no contact, you are so exhausted, and the baby has taken … everything from you. But when you see it on the video, it isn’t like that at all, the baby hasn’t taken everything from you, he has given you lots. It is a feeling of joy and of being a mother*” (mother with high depression scores; Vik & Hafting, 2009). Parents who were reluctant to accept the practitioner’s information and reassurance could believe what they saw in the video, as opposed to when they were told information, and it reminded them of the practitioner’s comments (e.g., Auge, 2005).

While child behaviours were discussed with warmth, parents’ own behaviour seen on video was rarely mentioned. One mother in child welfare services remarked positively on noticing her own smiling to her child in the video which she did not notice herself doing in real life (Bergsund et al., 2024). Parents whose child had a difficulty especially focussed on discussing the child’s behaviour from the videos rather than the interaction between them.

##### Subtheme 2: Video as an Agent for Change

Parents described behavioural changes that occurred as a result of the process of reviewing the videos together with the practitioner in the intervention. Some verbalised their own enhanced metacognitive and creative thought process, which helped them regulate and modify their own reactive and habitual tendencies: “*It was eye-opening because before it was, ‘Oh, okay, well she’s just putting that on wrong. I’ll show her the right way to do it’. But then … actually no, she’s exploring every element of it, she does that with everything. Why haven’t I picked that up before?”* (adoptive parent; Dugmore et al., 2022). Similarly, a Native American mother reflected on ‘self-correcting’ their parenting responses: “*I see it when I get frustrated with him. I have to look back and think, ok this is the wrong way. I need to calm down. He’s just a little boy. Let him do things, and let him help*” (O’Leary et al., 2022). These behavioural changes seem to be rooted in an increased capacity for keeping in mind the child as a separate mentalising being: “…*it just seemed like she was a baby who sleeps and drinks and now I can notice that she is more responsive and fun…noticing that all of a sudden she starts to become a little person*” (parent with difficulties managing their child’s behaviour; Iles et al., 2017).

In several studies, parents reported that a particularly impactful lesson from the VF was learning to wait more for the infant to take the lead. This simple practice facilitated the parent’s reflective process in-the-moment and allowed the interactive dynamic to shift to a more child-centred one: “*I think being forced to slow it down and watch their reaction carefully and let them learn it at their speed is something I really took away”* (father with difficulties managing their child’s behaviour; Williams, 2017). Parents who participated in a more directive ‘coaching’ intervention also seemed to show hints toward increased reflective functioning in the reiterative VF process: “*It was just really helpful to review videos and actually see [Cece] responding, and seeing myself and learning... how we could carry on to the next session*” (mother of young child with communication delay; Ousley et al., 2022).

Parents with mental health and psychosocial difficulties also described how their changed perceptions from the VF led to enhanced awareness and responsiveness, as encapsulated by quotes from these authors: “*A mother described how the discovery of her son’s contact-initiative was decisive for her fast response […] Other times he needed active response from her to regulate emotions or states. She described how she adjusted her actions to the child’s expressions and needs. This mother reflected on the child’s inner state”* (Gill et al., 2019); *“Even a parent who said that she had not truly learned anything new that the guidance had strengthened her sense of the meaning of the minutiae of the interaction”* (Simhan et al., 2021b).

#### Theme 3: The Practitioner’s Skill and Role in Creating a Safe Space

The practitioner-parent relationship was highly valued across the range of sample populations. Parents viewed the practitioner as providing a non-judgemental space to talk about their feelings and for containment, which enabled trust and collaboration to develop. The practitioner’s skill in putting the parent at ease was pivotal to getting past the uncomfortable feelings identified in the first theme when filming and reviewing interactions: “*She was really considerate when she did the filming… The very first session was quite nerve-racking. She was just great, I mean, how she just really kind of made me almost forget about it”* (parent from socially disadvantaged community; Chakkalackal et al., 2021).

The (positive) feedback that the practitioner provided to the parent was also perceived as powerful; it reassured parents that they were doing *“the right thing”*. Some parents participated in the intervention specifically to receive ‘expert’ reassurance, even if they expressed no particular concerns. Perceptions of the practitioner seemed integral to the success of VF. One father articulated: “*To take pauses in the film is incredible important, (…) to get one or two minutes to reflect on it. (…) You challenged me; why do you think he had that facial expression? What do you think he is thinking about? It is really quite positive to be challenged like that because one begins to reflect on what he expresses in different situations*” (father at high psychosocial risk and interactive difficulties; Gill et al., 2019). In this example, both the therapeutic space (or time) and the practitioner’s challenging questions were valued.

Many parents felt that the practitioners’ observations and skills were integral to facilitating change. Referring to mothers with a child with possible emerging autism, Amsbary et al. (2021) reported: “*All parents agreed that the interventionist and the relationship they had with her contributed greatly to their ability to implement the intervention*”. The authors cited quotes from parents such as: “*the first teacher [to help] me understand my son*,” and “*She was very supportive and was very good at explaining what we’re looking for*”. Parents felt that the practitioner was good at noticing subtleties and had the verbal skills to articulate them. An adoptive parent offered an example which also illustrates a mentalising stance: *“… just noticing things, seeing things that [the VIPP intervener] noticed that I wouldn’t have noticed … like my son putting his hand on my knee when he was playing with something he was enjoying … and that was just a subtle way of saying, ‘This is lovely, I’m enjoying this, I want to share it with you’*” (Dugmore et al., 2022).

By contrast, one mother felt alienated by the practitioner’s stance: “*She is so extremely positive … everything is, like, I feel she is looking at everything through rose-tinted glasses. She is either being very professional or just a very happy person, at peace with herself. Which I am not*…” (mother with mental health difficulties; Simhan et al., 2021b). This quote illustrates how the relationship with the practitioner is absolutely integral to the effectiveness of the intervention. This mother perceived the practitioner’s non-attunement as jarring or affronting. She described the practitioner as “ignorant” and patronising, but continued participating in the intervention.

#### Theme 4: The Approach Was Too Intangible, Inflexible, Positive, Unclear

Most interventions (when information of the intervention was provided) were described as client-led, strengths-based or non-directive, a guidance approach that suits the VF technique as video serves as the catalyst for change. This approach was viewed positively by most participants across studies. However, a point that arose across some studies was that some parents expressed varying degrees of dissatisfaction with participating in a VF intervention. For various reasons, the approach did not provide them with what they had expected or hoped for. For these parents, participation was a frustrating, challenging or confusing experiences, and they felt they had not gained enough of tangible value by participating: *“I don’t really get a sense that very much was suggested it felt very non directional. So in terms of us having strategies to change… I didn’t get them and still haven’t got them.”* (father of infant with behavioural issues; Williams, 2017). One mother with mental health difficulties felt even more strongly, describing the non-directive approach (“*A lot of ‘how do you feel about this?’ and ‘what do you think when you see that?’ kind of stuff…)* as “ridiculous”, “obvious” and insulting (Simhan et al., 2021b).

Some parents expressed that they wanted advice and training in parenting strategies to support their child’s needs, which were not forthcoming with a client-led approach. Coaching methods were preferred by some, especially to manage children’s high distress and challenging behaviours. When asked how the programme they received could be improved, many parents detailed the need for more clarity about the intervention and potential outcomes from the practitioner to reduce parents’ confusion and fear of being criticised. Both intervention completers and non-completers in O’Leary et al. (2022) study of Native American families felt a need for clearer and more structured instructions, particularly around the videoing: “*I raised all my kids. I know how to play with my kids. I know how to play with my grandkids. I just didn’t know what I was supposed to do [during video].”*

For some parents, the structured approach utilised in the intervention was too rigid and did not address the situations that they wanted help with: “*It might have been useful to catch [infant’s name] displaying more challenging behavior that could be talked through in future feedback… For example, she struggles to brush her teeth and go to bed and it would have been great if you could have filmed those times*” (parents of toddlers with behaviour difficulties; Iles et al., 2017). Of fathers struggling to manage their infant’s behaviour, Williams (2017) summarised: “*Fathers typically spoke about the manualized approach limiting their ability to discuss their families’ individual concerns (e.g., temper tantrums) and that this sometimes felt frustrating and inflexible*”.

 However, based on one of the deeper analyses, the challenge for some parents may not be the non-directive nature of the approach, but that the positive stance taken can be ‘too’ confronting and dysregulating to parents when it is so discordant with the parent’s internal views of themselves. Of parents with mental health difficulties, the authors offer insight into this process: “*A central topic was the positive stance with its predominant focus on supportive interaction. Most parents had become distinctly aware of this because it collided with their negative expectations and felt new and even upsetting. They not only had negative ideas about themselves as parents that made it difficult to accept a positive stance but also felt that the problems in the interaction should have received more attention. Should the guidance not also inform them about what they did wrong? Could a positive review be trustworthy*?” (Simhan et al., 2021b).

#### Theme 5: When the Intervention Is Over: Positive Change and Generalisation

Parents across many studies outlined several areas of positive change from taking part in the intervention. They felt better able to recognise and understand their child’s cues and needs (e.g., Elliott, 2020), had a better understanding of how to play and enjoy interacting with their child and noticed more about their child’s developments (e.g., Cleary et al., 2022). Perceived benefits extended to psychological changes within themselves and generalised to other relationships outside of the parenting context. Some parents discussed experiencing improved self-esteem and confidence. Many discussed a renewed sense of valuing the importance of spending quality time as a family. For some, the act of participation itself helped to prompt beneficial discussions with partners, in turn improving their relationship.

A lasting increased desire to connect with the child was often described: “*My behaviour has changed in playing with her, to want to play with her, to be gentle, to bring out the toys and take them away calmly. I really learnt a lot*” (parent with difficulties managing their child’s behaviour; Iles et al., 2017). Of fathers of children with behavioural difficulties, Williams (2017) described that “*Many fathers said that since the intervention they understood their child’s communication more and were looking out for their child’s nonverbal communications*”.

Their intervention experience and learning seemed to increase parents’ capacity for reflection and empathic understanding that can apply to other relationships, especially with their older children: “*She kind of helped me to see the importance of spending time with my other children […] she gave me ideas. So it has improved, I would say, my relationship with my [older] son in particular because he was the one that got the least of my time*” (parent from a disadvantaged neighbourhood; Chakkalackal et al., 2021). A parent with chronic mental health difficulties reflected deeply: “*And I can use it in other situations in my everyday life, for example when I help my oldest son with his homework. In situations like that, I can see myself sitting there and I very nearly feel like my own mother. Like it’s me and my mother sitting there with my homework, and she is telling me what to do without explaining anything. And now I act differently when I am helping my son*” (Simhan et al., 2021b).

By contrast, few parents discussed specific changes in the child’s behaviour as a result of parental change, though it may have been too soon for parents to notice, and no studies conducted longer-term follow-ups. One parent of a child with language impairment relayed language improvements remarked on by a school teacher (Senent-Capuz et al., 2022).

#### Theme 6: Parental Engagement and Involvement: Barriers and Enhancements

While VF interventions varied in task load between sessions, typically parents are encouraged to implement behavioural changes in the form of focussed activities and/or within daily routines, since the sessions themselves tended to focus on video reviewing and reflective discussions. However, parents in nearly all studies cited a lack of time to fit these activities in around busy family schedules. Those studies that delved into this further found that the issue of time was combined with other challenges associated with schedules, other demands, children’s willingness to engage in focussed time and time to understand and digest the intervention content. Parents with a child with possible emerging autism found daily practice impractical, not only due to limited time in the day but also because it depended on children’s unwilling to participate (Amsbary et al., 2021).

Other commonly cited barriers to participation are shared across all parenting interventions, including lack of childcare, multiple children, managing competing demands, and travel. However, as attending sessions involving VF does not directly involve the child except for recording parent–child interactions, even having one child attending creates distractions. Addressing the need to travel, parents who received home-based intervention reported feeling more comfortable, relaxed and preferring this to a clinic setting, particularly new parents (e.g., Cleary et al., 2022). For one intervention non-completer, time and competing daily demands from participating signified a lack of perceived relevance and benefit: “*You just sit there to interact with your child for an hour. That’s not the way I do it at home. I’ll set something down. When he gets busy, I’ll get up and try to do something else because there’s always a ton of other things to do in the house*” (Native American families, O’Leary et al., 2022).

To benefit optimally from the intervention, some parents expressed needing more time to take in content. For example, parents who participated in one programme, comprising ten one-hour home sessions delivered by child welfare services, reported that it was too brief to grasp and absorb all the content (Bergsund et al., 2024). Following an intervention of eleven sessions, one mother of a child with communication difficulties reported: “…*because we liked it so much that we would like to keep on learning much more about this. But it is fantasti*c” (Senent-Capuz et al., 2022).

While it is unclear how much of the time issue is specific to the VF component, some parents cited wanting more time to review videos, or being unable to remember the feedback. A phone app was suggested to enable access to videos (reviewed during sessions) in their own time to digest the information (Chakkalackal et al., 2021). A further recommendation across some studies was including additional caregivers and family members in the intervention to encourage consistent parenting and greater ecological validity, though the practical challenges in coordinating schedules are recognised. A few parents recommended sharing information with significant people in the child’s life.

## Discussion

In this first review of parents’ experiences of VF interventions in the early years, we synthesised seventeen studies of 157 caregivers with a wide range of characteristics, including parental mental health difficulties, child behavioural or communication difficulties, and families at psychosocial and relational risk. Ten studies were published since 2021, showing an uptick in interest in exploring parents’ experiences to inform clinical developments and practice. Individually, many of the studies were restricted in scope and involved very small samples, and the number of studies of each population group was very small. Thus, combining these efforts into a thematic metasynthesis enabled us to provide a fuller picture, emphasising commonalities in experience described by primary caregivers with infants and young children across a range of programmes and contexts. Taking a perspective across multiple studies allowed us to weave a broadly chronological narrative of the parent’s journey through the VF intervention process (Fig. 2). We identified issues and themes that were more pertinent to particular groups, and challenges at specific points as well as for particular parents.

The parent’s journey through the VF intervention process across different groups was mostly experienced as positive, and often transformative. The range of interpersonal (e.g., improved relationships with other family members) and intrapersonal benefits (e.g., improved self-confidence) expressed by parents often went beyond the intended outcomes that are commonly measured in evaluation studies. Such overall positive parental experiences align with the positive relational outcomes reported in quantitative reviews of VF parenting interventions (O’Hara et al., 2019). Of particular interest, we found that parents across studies who had identified bonding issues or who lacked parenting confidence particularly valued the strengths-based, reflective and empowering nature of VF. This somewhat challenges previous meta-analytic findings which suggest that parenting interventions are less effective in changing attitudes when the parents are a high-risk group (Bakermans-Kranenburg et al., 2003) and that VF interventions are ineffective at improving parental stress and anxiety (O’Hara et al., 2019). Thus, the findings of our metasynthesis complement meta-analytic approaches when making informed decisions about the suitability of specific intervention approaches.

Perhaps the richest theme that came through was the great impact that reviewing videotaped parent–child interactions had on parents. The use of video was reflected on by authors as essential to its success. By noticing ‘from the outside’, focussed attention on the video and emotional arousal associated with viewing oneself was important, as emphasised by Aldred et al. (2018). Parents imbued positive intentionality from their child toward *them*, and this changed how they perceived their child and relationship. For mothers and fathers across diverse sample populations, but especially those with mental health difficulties and/or struggling to bond with their infants, the video provided the validation they needed. The infant’s gaze (to the parent) held special meaning, and was described as joyful, surprising or reassuring.

Critically, the most change seemed to be reported by those parents who expressed their reflective capacity as enhanced as a result or who reported more regular practice in their daily interactions. These parents gained new insights into the child’s mental world through the VF process, which disconfirmed their previously held beliefs. After receiving the VF, some described resisting enacting reactive or ‘corrective’ behaviours, instead waiting for the child to lead, and/or a new enjoyment in seeing what happens next. Furthermore, this newfound or enhanced reflective practice, empathy and/or valuing of communication was found in our metasynthesis to later extend to other relationships – a benefit that parents value, particularly those with multiple children, more than some practitioners realise (Doria et al., 2014). However, these parents who enjoyed greater levels of metacognition may also be better able to articulate such outcomes.

However, some parents expressed varying degrees of negativity about participating in a VF intervention. The extent to which issues were specific to the VF process itself is often unclear in studies. While our first theme combined negative feelings elicited by the VF process itself (e.g., being videotaped and feeling pressure to ‘get it right’) with the process of overcoming those feelings to gain insights and benefits from the process, because these codes frequently co-occurred, it is unclear from studies whether some parents never overcame this initial stage in the early sessions. While experiences were rarely described as wholly negative, the more negative comments seem to be shaped by the parents’ prior expectations about the intervention approach and goals, how closely they match their own perceived needs, their beliefs about the impact of their own behaviour on the child’s, and their willingness to take a reflective stance and value reflective discussions. A non-directive approach was perceived as lacking benefits for some parents’ needs; they felt frustrated about ‘not gaining something of value’ from reviewing video clips, especially with having to take the videos in the first place. Other issues align with previously identified barriers to parenting interventions generally, including feeling that the goals for the programme were not met, fear of being judged, and competing demands on time (Jukes et al., 2024). While the more negative experiences and perspectives were not as common as positive ones, scope to provide general opinions about the VF technique were likely restricted in some studies.

### Clinical Implications

Parents’ experiences suggest that the use of personalised video feedback can be very powerful for bringing about psychological and behavioural changes in how parents relate to and understand their child and other relationships. Increasing accessibility and flexibility to allow parents to revisit video and learning points, and to allow other family members to be involved in the VF experience, is favoured. Combining home visits with online facilities can also help make this a more immersive experience. However, the importance of offering childcare, particularly with VF approaches, should not be overlooked, and community settings may be most appropriate in order to offer convenient group-based childcare to families.

The practitioner’s role is more critical than the use of VF, of which a large part must involve preparing to align parent expectations and foster a collaborative, strengths-based approach. In the preparation phase, open discussions about the VF process and each person’s roles within it are essential (Klein et al., 2021). Emphasising the non-directive approach and managing expectations about coaching and advice can prevent misunderstandings. Common ground regarding the inseparability of parental and child behaviours from relationship dynamics must be established. Video reviewing may present a ‘reality’ discordant to parents’ perceived one, so the practitioner’s meaning-making must be managed in ways that make sense to the parent to avoid misattunement (Vik & Rohde, 2014). For those who struggle with the use of video, initial recordings can focus on the infant, keeping the parent out of the frame. Practitioners can experiment with alternative approaches, such as allowing parents to submit self-recordings or use audio recorded or live observations, which may reduce anxiety and performance pressure (O’Leary et al., 2022).

One-to-one VF interventions are a highly involved and intense approach. Barriers and resistance reported by some parents suggest that some parents are not yet ready to commit to such a personal and emotive process. These issues are likely under-reported due to study biases (e.g., high self-selection, lack of inclusion of non-completers, practitioner-interviewer dual roles). For those focused on enhancing relational outcomes, Wittkowski et al.’s (2025) review of non-VF attachment-based interventions identified forty studies covering sixteen manualised programmes, of which four fifths had reported significant positive relational change compared to pre-intervention or a control group. Of those with the most promising trial evidence, *Circle of Security Parenting,* like VF approaches, also includes video use and reflective discussions, but within a group setting and includes discussions about parents’ own attachment experience and their behaviour towards their child. For parents who struggle with the behavioural level at which VF operates, COS-P allows for broader discussions focussed on parents’ own past (Helle et al., 2022) While videos were usually of other parents in the group, one study suggested that they easily identified with reviewing other parents’ videos and related them to their own experience (Jonsdottir & Coyne, 2016). For parents with younger children, Wittkowski et al. (2025) also identified *Attachment and Biobehavioral Catch-Up (ABC)* and *Minding the Baby* home visiting programme as having the most promising evidence, involving in-the-moment addressing of parent–child interactions and reflective discussion. For those parents who may struggle with reflective parenting approaches. Bergsund et al. (2024) suggests an alternative for delivering VF in a non-directive format, but with emphasis on observed behaviour rather than on reflection *per se*.

### Future Research Directions

Careful evaluation of evidence-based interventions, adapted for specific communities and needs, is critical to the long-term success and sustainable implementation of VF programmes. Thus, efforts are needed to reduce potential sources of bias in future qualitative studies of parental experience by including intervention non-completers, ensuring interviewer independence from the therapy process, and providing more complete reporting, including of the sampling process or why they sought the views of those particular parents, and how long post-intervention parental views were sought. Parallel qualitative studies as an adjunct to primary outcome studies would provide value for informing clinical practice and intervention development, and for developing a theory of change through the VF intervention process. Future studies could qualitatively explore ‘critical junctures’ in the VF journey, such as when deciding whether to participate, overcoming negative thoughts and feelings roused by the VF process, and generalising realisations about one’s own child to other relationships. For those wanting to gain parental feedback via a quantitative approach, the themes in this review can provide a framework to incorporate a broader scope of topics in a questionnaire design.

Addressing systemic barriers with sufficient resourcing is imperative to provide more equitable opportunity for those who stand to benefit most. Incorporating the parents’ perspective into the design or adaptation stage, in a co-constructed process, would be in keeping with a non-directive intervention’s approach and could enhance future recruitment and retention. Research involving parent and practitioner perspectives is needed to better understand the degree to which systemic and perceived barriers can be ameliorated by building in more flexibility (e.g., with session times, home-based sessions), personalising the sessions more (e.g., the degree of reflection expected, some online delivery, allowing review of videos outside of sessions), and clarity (e.g., preparing parents for what to expect while normalising their feelings of anxiety, carefully scaffolding instructions, clarifying the parent’s role). Cultural issues were rarely addressed in our reviewed studies.

### Study and Review Limitations

The studies reviewed are limited in several ways that affected our synthesis. Study quality was highly variable, reflecting their often pilot nature and biased design. The dominance of studies testing adaptations and focusing on underserved populations meant the views that were captured may reflect less typical implementation relative to what happens in practice. Furthermore, the practitioner in some studies was also the interviewer. In all VIG and Marte Meo studies, this was either the case or interviewer independence was not declared. The parent develops a relationship with the practitioner over time and may understandably feel reluctant to disclose negative thoughts about a process in which the practitioner has invested. Some interventions were delivered within services (e.g., mental health, child welfare) in which the participant was a service user, again likely affecting what parents reported. Participant also heavily self-select, as parents who had a positive experience would be more motivated to discuss it. The views of intervention non-completers are barely known, since only one study included non-completers. Finally, the practical barriers (e.g., time limitations) and the discomforts of the early sessions, which were among the most common themes in our metasynthesis, may have been considered more acceptable by parents to discuss as they avoid being critical of the intervention or practitioner.

There are additional shortcomings and considerations in the review itself. Firstly, only English language studies were reviewed, which may limit representation. All reviewed studies had been conducted in Western countries; only one study involved a minoritised community (O’Leary et al., 2022). Secondly, the exploratory nature of the review meant that study quality was highly variable and data saturation likely not reached in several studies. Yet reports of small samples tended to be richer and more extensive, so quotes from these sources were more prolific. With this awareness, we attempted to maintain balance in our analysis by including quotes from a range of samples and quoting author interpretations (not only participant quotes). Thirdly, variation in intervention characteristics reduced study comparability, despite a VF focus and one-to-one discussions with a professional. These variations include the extent to which the approach was practitioner-led, the theoretical underpinning (e.g., whether it was explicitly attachment-based), the number of sessions and intervention duration, and the presence of non-VF elements. Fourthly, as the success of interventions may lie in how well tailored they are to specific communities (Law et al., 2009), parental views of critical elements of individual programmes will have been lost in our bid to synthesise common themes across studies for diverse populations. Finally, while regular reflective practice and team discussions were put in place to minimise bias, the research team’s experience as mental health professionals and academics in perinatal psychology and VF interventions may have affected our interpretations of the data.

## Conclusion

Parental acceptability of VF parenting intervention usually relies on crude measures, such as completion rates and satisfaction ratings based on predetermined scales, which tell us little about degree of parental involvement, the change process, and unanticipated benefits and issues. In recent years, researchers have been increasingly interested in obtaining parental perspectives by examining their lived experiences of the intervention process across a range of well- and lesser-known VF programmes. As lived experience research has become more integrated into the research process to guide clinical developments, these studies are increasingly valued for improving the quality of reviews, which can be particularly informative to practitioners (Beames et al., 2021). Ten of the seventeen studies reviewed were published since 2021. Based primarily on interviews and focus groups, we described commonalties and divergences in the parent’s journey through VF interventions, including the multifaceted ways in which parents experience VF and the change process, and why some parents struggle to benefit from the approach. In conclusion, parental qualitative experiences are a valuable complement to outcome-based evaluations, particularly when conducted with the same sample, to inform ways to optimise parental readiness for the VF approach, although biased design and lack of reporting completeness need addressing in future studies.
